# Activated coagulation time *vs.* intrinsically activated modified rotational thromboelastometry in assessment of hemostatic disturbances and blood loss after protamine administration in elective cardiac surgery: analysis from the clinical trial (NCT01281397)

**DOI:** 10.1186/1749-8090-9-129

**Published:** 2014-09-17

**Authors:** Mate Petricevic, Bojan Biocina, Davor Milicic, Lucija Svetina, Marko Boban, Ante Lekić, Sanja Konosic, Milan Milosevic, Hrvoje Gasparovic

**Affiliations:** Department of Cardiac Surgery, University Hospital Center Zagreb, University of Zagreb School of Medicine, Zagreb, Croatia; University of Zagreb School of Medicine, Department of Cardiovascular Diseases, University Hospital Center Zagreb, Zagreb, Croatia; Department of Cardiology, University Hospital “Thalassotherapia Opatija”, Medical School University of Rijeka and Osijek, Opatija, Croatia; University of Zagreb School of Medicine, Department of Anesthesiology, University Hospital Center Zagreb, Zagreb, Croatia; Andrija Stampar School of Public Health, University of Zagreb School of Medicine, Zagreb, Croatia

**Keywords:** Rotational thromboelastometry, Hemorrhage, Cardiac surgery, Activating coagulation time, Transfusion, Bleeding management

## Abstract

**Background:**

Excessive bleeding after cardiopulmonary bypass (CPB) is risk factor for adverse outcomes after elective cardiac surgery (ECS). Although many different point-of-care devices to diagnose hemostatic disturbances after CPB are available, the best test is still unclear. The study aim was to compare the accuracy of hemostatic disorder detection between two point-of-care devices.

**Methods:**

We enrolled 148 patients (105 male and 43 female) undergoing ECS in a prospective observational study. Rotational thromboelastometry (*TEM,* with InTEM test), and Activated coagulation time (ACT) measurement were performed 15 min after protamine administration. The cohort group was divided into two subgroups according to occurrence of excessive postoperative bleeding. Endpoints were defined in two ways: as total amount of chest tube output (CTO) and blood product transfusion requirements.

**Results:**

Total amount of CTO value of 1507,50 mL presented 75th percentile of distribution, thus cut-off value for bleeder category. InTEM parameters, but not ACT, correlated significantly with CTO. InTEM parameters with the strongest correlation to CTO were tested for accuracy in predicting excessive postoperative bleeding using ROC analysis. InTEM A 10 value of 38 mm, InTEM A 20 value of 49 mm and InTEM A 30 value of 51 mm delineated bleeding tendency. Patients with total amount of CTO exceeding 75th percentile were more frequently transfused with fresh frozen plasma (51.4% vs. 9.9%, p < 0.001), fibrinogen concentrate (21.6% vs. 2.7%, p = 0.001) and platelet concentrate (13.5% vs. 0.9%, p = 0.004).

**Conclusion:**

Our study showed that InTEM test, but not ACT is useful in prediction of bleeding tendency after protamine administration following weaning from CPB. InTEM test could be used as a first line test in screening of possible hemostatic disorder following protamine administration.

**Electronic supplementary material:**

The online version of this article (doi:10.1186/1749-8090-9-129) contains supplementary material, which is available to authorized users.

## Background

Excessive bleeding after CPB continues to be an important cause of morbidity and mortality [1]. Dixon B et al. reported chest tube output (CTO) as the strongest independent predictor of mortality [2]. However, the question how to predict and prevent excessive bleeding due to hemostatic disturbances remains challenging. Therapeutic approach to surgically or coagulopathically-induced bleeding is quite different. In addition to, conventional coagulation tests are not able to predict postoperative bleeding [3, 4]. Thus, bedside suitable devices capable to identify hemostatic disturbances after weaning from cardiopulmonary bypass (CPB) are desirable. Identifying hemostatic disorder after CPB may make the important differentiation between coagulopathic (nonsurgical) bleeding requiring procoagulant blood components transfusion and surgical bleeding requiring surgical intervention.

The Activated Coagulation Time (ACT) was first described by Hattersley in 1966 and is essentially a point-of-care test of coagulation that is used to monitor the anticoagulant effect of unfractionated heparin (UFH) in patients on bypass surgery [5]. The ACT first came into clinical use in the mid-1970s to guide the administration and reversal of heparin during CPB procedures. Although originally proposed as a routine pre-operative screening test - it is now used almost exclusively for monitoring patients on CPB. The test is now more commonly performed using a fully automated technique with several devices available in which the end point i.e. clot formation is recorded electronically – the principle, however, remains the same as described by Hattersley many years ago [5]. ACT is the most common intraoperative point-of-care hemostatic test to confirm profound anticoagulation during CPB and adequacy of heparin-protamine neutralization after weaning from CPB [6]. Although the most commonly used hemostatic point-of-care device in cardiosurgical procedures, the ACT is the least sensitive hemostatic test to detect residual heparin anticoagulation [6], as well as other hemostatic disorders which may lead to excessive bleeding after CPB. Although ACT is regularly used for assessment of heparine-protamine neutralization management adequacy and assesses intrinsic pathway of coagulation, its reliability in predicting hemostatic disorder resulting in excessive bleeding remains elusive. The best point-of-care test to diagnose hemostatic disorder immediately after CPB, is still unclear. Rotation thromboelastometry (TEM) performed on whole blood samples provides information on the contribution of fibrinogen and platelets to clot formation, however, the precise relationship between TEM values and postoperative bleeding still remains unclear.

This study sought to compare and determine the accuracy of intrinsically activated rotational TEM test (InTEM test) variables and ACT in detection of excessive bleeding and blood products transfusion requirements after CPB. This study has been presented during 23rd World Congress of the World Society of Cardio-Thoracic Surgeons and published in supplement issue of the Journal of Cardiothoracic Surgery [7].

## Methods

This study presents retrospective exploratory analysis of prospectively collected data within research project approved by the Ethics Committee of the University Hospital Center Zagreb and registered at the Clinical-trials.gov website (Identifier NCT01281397). The purpose of this study was prospectively defined. Clinical trial NCT01281397 was designed in prospective observational fashion. The aim of trial was to assess possibility of point-of-care hemostatic devices (rotational thromboelastometry and multiple electrode aggregometry) to predict excessive bleeding in elective cardiac surgery. Parameters of rotational thromboelastometry and multiple electrode aggregometry were obtained perioperatively at three time points and respective values were correlated with observed key endpoints such as CTO and transfusion requirements. Initially, a trial was supposed to recruit 400 patients. During study period, interim statistical analysis was performed after approximately every 50 patients. After 148 patients enrolled, interim analysis revealed positive results in regard to primary hypothesis that point-of-care tests for assessment of platelet function and viscoelastic blood properties predict bleeding in cardiac surgery patients. Thus, due to fact that positive results confirmed primary hypothesis after 148 patients were recruited, we decided to terminate study earlier.

### Patient selection

After approval by the local School of Medicine and University Hospital Center Ethics Committee and written informed consent, 148 consecutive patients (male, n = 105 (70.9%); female, n = 43 (21.9%)), scheduled for elective cardiac surgery (ECS) procedures requiring CPB between July 2010 and January 2011 were recruited. Excluding criteria were: age younger than 18 years, urgent procedure, administration of antiplatelet agents other than aspirin and clopidogrel, patients with inaccurate antiplatelet therapy administration documentation, patients with off-pump cardiac surgical procedure, and patients requiring surgical exploration for excessive bleeding due to obvious surgical bleeding with a bleeding vessel identified.

### Perioperative management

All patients had the same anesthetic and perfusion teams, and were admitted at least 1 day before surgery. Surgery was performed by using a standard technique. All measurements were performed by research fellow, not directly included in treating patients. The nurses, anesthesiologists, and surgeons managing the patient's care were unaware of InTEM results. Surgery was performed in a single unit with standard surgical techniques. Surgical bleeding was controlled with diathermy and bone wax.

The patients received diazepam and morphine 30 min prior to the induction of anesthesia. Endotracheal tube, urinary catheter, as well as radial artery and pulmonary artery catheters were inserted. The anesthetic regime included induction and maintenance of anesthesia with midazolam, fentanyl and pancuronium bromide. This was coupled with sevoflurane inhalation. The initial ventilator settings included a tidal volume of 8 ml kg_1, and a respiratory rate of 12 breaths per minute. Typically, the FiO2 was set at 50%. Cardiopulmonary circuit consisted of the Medtronic Affinity Trillium membrane oxygenator, venous reservoir and PVC tubing (Medtronic, Minneapolis, MN, USA) and a Stoeckert III roller pump (Stoeckert, Munich, Germany). Flow at 2.2 l min-1 m-2 and mean blood pressure of 60 mmHg were targeted. Heart was arrested with cold blood cardioplegia. Before CPB, 300 U/kg of heparin were administered. An additional 10000 units of heparin were included in the bypass circuit. Heparin dosing during CPB was based on ACT results repeated every 30 minutes. Systemic heparinisation aiming at an activated clotting time >480 s was used, followed by full reversal with protamine after decannulation. An additional heparin bolus dose (50 to 70 U/kg) was administered if an ACT value was less than 480 seconds. At the conclusion of CPB, the total heparin dose was antagonized using 1.0 mg of protamine for each 100 U of heparin. If the ACT exceeded value of 150 sec after protamine administration, additional dose of 50 mg of protamine was administered. 15 minutes after protamine administration, a blood sample was obtained to determine both the ACT and InTEM tests. A dose of 1 g tranexamic acid was given at the induction of anesthesia and after protamine administration. Inotropic support was initiated in order to maintain a cardiac index greater than 2.2 l min-1 m-2. Weaning from CPB was initiated once the patient’s rhythm had stabilized and normothermia had been achieved. Cardiotomy suction returned blood to the CPB circuit. Packed red blood cells (PRBC) were transfused if hematocrit was <20% during CPB and < 25% after terminating CPB, or when significant bleeding was obvious. Volume replacement in the intensive care unit was administered as deemed necessary by the attending physician using hydroxyethyl starch 6% 130/0.4 and lactated Ringer's solution, PRBC's were transfused if deemed necessary by the consultant anesthesiologist.

Fresh frozen plasma (FFP) was given in cases showing prolonged prothrombin time (less than 45%), or according to clinical decision by the consultant anesthesiologist. Fibrinogen concentrate and platelet transfusion were administered in cases of excessive bleeding at discretion of consultant anesthesiologist. We do not have specific cut-of values for transfusion of fibrinogen concentrate as well as platelet concentrate. If patient was exposed to antiplatelet drugs in close proximity to surgery, transfusion of platelet concentrate was considered. However, in cases where coronary revascularization has been performed, administration of platelet concentrate was avoided whenever possible and fibrinogen concentrate was used as a first line therapy instead.

### Blood sampling

Blood samples for InTEM and ACT measurements were obtained 15 minutes after protamine administration via central venous port. For InTEM test blood was collected in 1.8 mL sodium citrate (0.109Molar/3.2% citrate concentration) Vacutainer^®^ plastic tubes. InTEM tests were performed in up to 20 minutes after blood withdrawal.

We used two *point of care* (POC) devices for hemostatic properties assessment.

### Modified rotational thromboelastometry (TEM)

TEM was performed according to the manufacturer' instruction using equipment and kits provided by TEM International GmbH, Munich, Germany. TEM provides a continuous measure of the clot firmness, whose amplitude is given in milimeters. Technical details of the TEM analyzer have already been described elsewhere [8]. Put briefly, by digital data processing, the following typical variables are obtained: clotting time (CT), the time from the start of measurement until the onset of clotting; Clot formation time (CFT), the time between the onset of clotting and the moment when the clot firmness reaches an amplitude of 20 mm; A 10, A 20, A 30 corresponds to the maximum amplitude of the curve after 10, 20 and 30 minutes, respectively. INTEM is a baseline test that uses an ellagic contact activator for analyzing the general cagulatory status of the patient.

### Activated coagulation time (ACT)

ACT measurements were performed 15 minutes after protamine administration, with the technique of Kurec et al. [9]. ACT was first described by Hattersley in 1966 [5] and is a point-of-care test that is used to monitor the anticoagulant effect of unfractionated heparin. We used Medtronic ACT Plus™ (Medtronic Perfusion Systems, Minneapolis, MN) a microprocessor-controlled coagulation instrument used for in vitro real-time clot detection during CPB. Medtronic ACT Plus has multiple testing applications. The High Range ACT (HR-ACT) is a functional evaluation of the intrinsic coagulation system. The endpoint of the test is clot formation. This is detected by the change in fall rate of which is detected by a photo-optical system. Clotting is initiated through surface contact with a kaolin activator. The test is performed on 0,4 mL of fresh whole blood per cartridge channel obtained by venous access lines. Each HR-ACT cartridge is supposed to be pre-warmed prior to blood insertion. After 300 seconds of incubation, clot detection begins. The analyzer timing range is from 6 to 999 seconds.

### Evaluation of bleeding

Blood loss after CPB was evaluated in two ways. To estimate bleeding extent after protamine administration we meticulously documented CTO until chest tubes removal. The volume of CTO was expressed in mL. The cohort group was divided into two subgroups according to occurrence of excessive postoperative bleeding. Patients were characterized as bleeders if their CTO exceeded 75th percentile of distribution. In addition to, transfusion requirements (PRBC in mL, FFP in mL, fibrinogen concentrate in grams and platelet concentrates in units) were documented as surrogate markers of bleeding extent. InTEM variables and ACT were correlated to extent of CTO as expressed in ml and to quantum of transfusion requirements for each blood product.

### Statistical analysis

The Kolmogorov-Smirnov test was used for evaluating the normality of distribution of all continuous variables. Correlation between CTO and InTEM parameters was evaluated by Spearman's correlation coefficient. Mann–Whitney U test was used to evaluate whether the medians on a test variables differ significantly between two groups. Chi-square statistic test was used to compare a frequency distribution of observed categorical variables between the two groups. Receiver operating characteristic curve (ROC) was constructed to assess the ability of TEM parameters to predict excessive postoperative blood loss [10]. A value of p < 0.05 was considered statistically significant. For statistical analysis we used MedCalc^®^ For Windows (MedCalc Software, Broekstraat 52, 9030 Mariakerke, Belgium).

## Results

**Figure 1:**
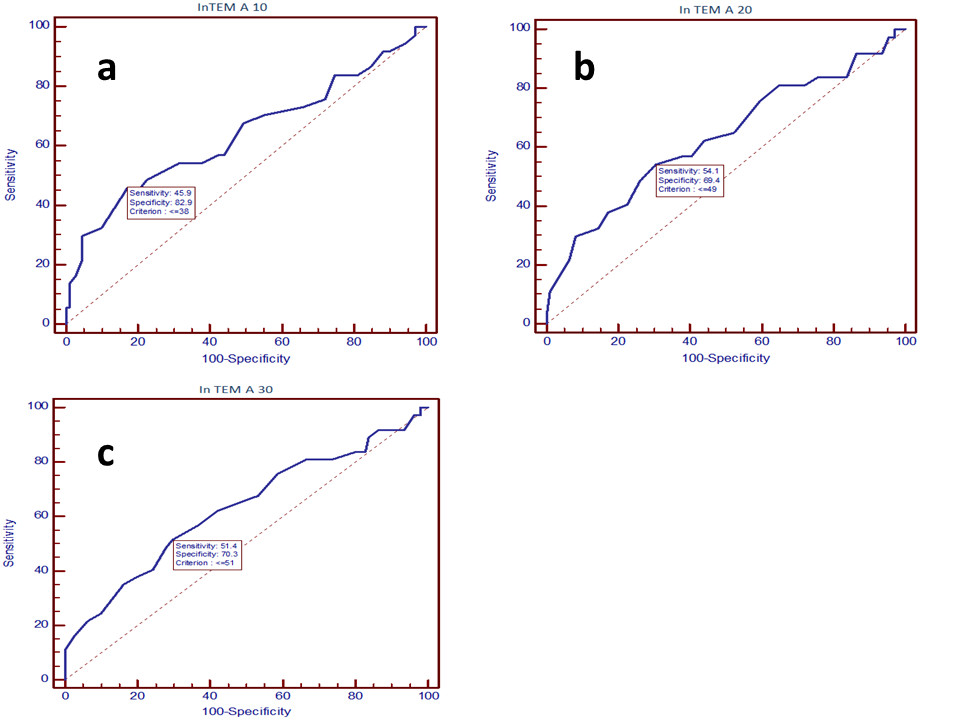
**Receiver operating curve analysis for excessive bleeding prediction using (a) InTEM A 10 test (AUC 0.636, p = 0.02) , (b) InTEM A 20 test (AUC 0.632, p = 0.02) and by (c) InTEM A 30 test (AUC 0.630, p = 0.02).**

All patients from sample were discharged from hospital. Study cohort demographic, surgical, and baseline routine laboratory findings are presented in Tables 1 and 2. Total amount of CTO value of 1507,50 mL presented 75th percentile of distribution, thus a cut-off value for bleeder category. No surgical reexploration was performed in study cohort. Patients in “Bleeder” category had longer CPB time (median, 116 vs. 95 min, p = 0.023) and significantly lower value of the lowest body temperature during CPB (median, 30 vs. 32 degrees of Celsius, p = 0.012). Patients with total amount of CTO exceeding 75th percentile were more frequently transfused with fresh frozen plasma (51.4% vs. 9.9%, p < 0.001), fibrinogen concentrate (21.6% vs. 2.7%, p = 0.001) and platelet concentrate (13.5% vs. 0.9%, p = 0.004). Differences in ACT and InTEM parameters of patient groups according to bleeding tendency are presented in Table 3. InTEM parameters, but not ACT correlated to total amount of CTO (mL) (Table 4). InTEM parameters with the strongest correlation to CTO were tested for accuracy in predicting excessive postoperative bleeding using ROC analysis. Accuracies of the InTEM A 10 (AUC 0.636, p = 0.02), InTEM A 20 (AUC 0.632, p = 0.02) and InTEM A 30 (AUC 0.630, p = 0.02) are shown in Figure 1.

**Table 1:** **Basic demographic, laboratory and operative data statistics of study cohort for continuous variables**

	Number of patients	Median	Interquartile range
Age (years)	148	66,00	56,50-73,00
Body mass index (kg/m^2^)	148	28,22	25,72-31,35
Body surface area (m^2^)	148	1,97	1,82-2,13
EURO score (%)	148	3,15	1,83-5,57
Ejection fraction (%)	148	58,50	49,00-65,00
Platelet count (x10^9^/L)	148	205,50	172,00-250,00
Fibrinogen (g/L)	148	3,85	3,30-4,55
Hemoglobine (g/L)	148	135,00	123,00-145,00
Hematocrit	148	0,41	0,37-0,43
International normalized ratio	148	0,98	0,94-1,05
Cross-clamp time (min)	148	67,50	50,00-94,00
Cardiopulmonary bypass time (min)	148	98,00	75,50-127,00
InTEM CT	148	194,00	173,50-215,00
InTEM CFT	148	126,00	100,50-176,50
InTEM alfa	148	68,00	62,00-74,00
InTEM A10	148	45,00	39,00-49,00
InTEM A20	148	53,00	47,00-57,00
InTEM A30	148	54,00	49,00-58,00
Activated coagulation time after protamine administration (sec)	148	138,00	128,00-147,00
Ventilation (h)	146	10,00	7,00-13,00
Troponin T at postoperative day 1 (μg/L)	147	0,74	0,40-1,50
Total amount of chest tube output (mL)	148	1000,00	660,00-1505,00
Lowest body temperature during cardiopulmonary bypass (degrees of celsius)	148	32,00	29,50-32,00

**Table 2:** **Basic demographic, laboratory and operative data statistics of study cohort for categorical variables**

		Count (N)	N (%)
Gender (Male)		105	70,9%
Hiperlipidemia		103	69,6%
Hipertension		123	83,1%
Diabetes mellitus		45	30,4%
Smoking habit		30	20,3%
Beta blockers		113	76,4%
Amiodarone		16	10,8%
Antiplatelet therapy		104	70,3%
Lipid lowering drugs		107	72,3%
Procedure type	Isolated CABG	84	56,8%
	Valve procedure	34	23,0%
	Combined procedure	18	12,2%
	Other	12	8,1%
Transfused		116	78,4%
Transfused fresh frozen plasma		30	20,3%
Transfused packed red blood cells		119	80,4%
Transfused Fibrinogen concentrate		11	7,4%
Transfused Platelet Concentrate		6	4,1%

**Table 3:** **Differences in activated coagulation time (ACT) and Intrinsically activated modified rotational thromboelastometry (InTEM) parameters of patient groups according to bleeding tendency**

	C.T.O. Overall (mL)	N	Median	Interquartile range	P (Mann–Whitney U)
INTEM CT	Non-bleeder	111	194,00	171,00-214,00	0.379
	Bleeder	37	198,00	182,00-221,00	
INTEM CFT	Non-bleeder	111	124,00	98,00-165,00	0.033
	Bleeder	37	149,00	103,50-207,50	
INTEM ALFA ANGLE (degrees)	Non-bleeder	111	69,00	64,00-74,00	0.016
	Bleeder	37	65,00	56,50-72,00	
INTEM MCF	Non-bleeder	111	55,00	50,00-59,00	0.017
	Bleeder	37	51,00	46,00-57,00	
INTEM A10	Non-bleeder	111	46,00	41,00-50,00	0.013
	Bleeder	37	41,00	36,00-48,50	
INTEM A20	Non-bleeder	111	53,00	48,00-57,00	0.016
	Bleeder	37	49,00	44,00-54,50	
INTEM A30	Non-bleeder	111	55,00	50,00-59,00	0.018
	Bleeder	37	51,00	46,50-56,50	
Activated coagulation time after protamine administration (sec)	Non-bleeder	111	138,00	127,00-145,00	0.179
	Bleeder	37	141,00	131,50-149,50

**Table 4:** **Correlations of activated coagulation time and intrinsically activated modified rotational thromboelastometry parameters with total volume of chest tube discharge (mL)**

	Spearman's correlation	Total amount of chest tube output (mL)
	Study cohort: 148 patients	
InTEM CT	Correlation coefficient	0,165
	P	0,045
InTEM CFT	Correlation coefficient	0,164
	P	0,047
InTEM alfa	Correlation coefficient	-0,199
	P	0,015
InTEM MCF	Correlation coefficient	-0,193
	P	0,019
InTEM A10	Correlation coefficient	-0,205
	P	0,013
InTEM A20	Correlation coefficient	-0,201
	P	0,014
InTEM A30	Correlation coefficient	-0,214
	P	0,009
Activated coagulation time after protamine administration (sec)	Correlation coefficient	0,053
	P	0,519

Blood sampling was performed 15 min after protamine administration.

When analyzing transfusion outcomes, InTEM CT parameter was shown to be significantly higher (Median 210 sec *vs.* 192 sec , p = 0.036) in patients transfused with FFP. The rest of InTEM parameters as well as ACT did not differ between patients divided with respect to FFP transfusion. InTEM CT parameter was significantly longer in patients transfused with fibrinogen concentrate, while the rest of InTEM parameters and ACT were not significantly different. Notably, no differences were observed in InTEM and ACT parameters when patients were divided according to platelet concentrate transfusion.

## Discussion

The best test to detect hemostatic disturbances and optimize hemostatic properties after CPB remains unclear. Conventional laboratory tests are unable to decompose multifactorial coagulopathy pathogenesis as described by Paparella et al. [11]. Considering the fact that immediate and precise diagnosis of the underlying cause of hemostatic disturbances, which result in excessive bleeding, is essential, we decided to compare ACT with another bedside suitable test, TEM with InTEM parameter. Our study showed that InTEM parameters, but not ACT, significantly correlated to bleeding extent following CPB. There are several advantages of InTEM test over ACT. (1) ACT fails to predict excessive bleeding. Observed correlations between ACT and CTO were not significant in our study (p = ns) which is in line with results recently published by Galeone et al. [12]. The role of the ACT as a monitor of profound heparin anticoagulation has also been questioned. Notably, Metz and coworkers found that maintaining a minimum ACT during CPB was not necessary if a 300 U/kg intravenous heparin dose was administered before CPB [13]. Worthwhile, a profound heparin anticoagulation can be easily confirmed with TEM using InTEM and HepTEM tests concurrently. (2) While Murray et coworkers [6] showed that ACT is not very sensitive to the low heparin concentrations that might be present after protamine reversal, Mittermayr et coworkers [14] described TEM as a valuable tool for heparin-protamine management. Using CT parameter, authors showed that TEM can be used to distinguish the effects of heparin excess from those of protamine excess [14]. Heparin rebound and incomplete heparin reversal are very common phenomena after cardiac surgery with CPB [12]. Recently, Galeone et al. [12] showed ACT not to be able to detect residual heparin activity, whereas thromboelastography analysis with and without heparinase allowed the diagnosis of heparin rebound [12]. However, no association was observed between heparin concentration, ACT, and thromboelastography parameters with postoperative bleeding and need for blood and blood components transfusions [12]. Contrary to, in our study InTEM CT parameter significantly correlated to the total amount of CTO. Although statistically significant, this correlation is relatively weak (p = 0.045), focusing CT parameter only to heparin-protamine management in cases of high InTEM CT values. (3) Hemostatic disturbances after CPB have multifactorial etiology due to many components of the hemostatic system involved in. TEM, but not ACT provides comprehensive insight into hemostatic properties, therefore allows possibility to detect the most dominant underlying cause of hemostatic disorder. (4). Spiess et al. reported thromboelastography guided hemostatic management to significantly reduce incidence of overall transfusion and mediastinal re-exploration due to excessive bleeding [15]. We believe that similar results could be obtained using TEM. However, such a hemostatic management should be evaluated through a prospective randomized controlled trial. In present study, observed InTEM parameters significantly correlated to total amount of CTO. Aside from heparin-protamine neutralization management, InTEM parameters provide possibility for targeted procoagulant blood component administration. Such a transfusion algorithm deems reasonable especially if InTEM parameters fall below cut-of values which delineate bleeding tendency according to the ROC curves. According to our results we propose following hemostatic management. In patients with high InTEM CT values we propose concurrent use of InTEM and HepTEM test with heparin-protamine management as proposed by Mittermayr et coworkers [14]. HepTEM test can be used to identify the presence of heparin and its effects on coagulation by comparison with the InTEM test. If bleeding occurs after InTEM-HepTEM heparin neutralization management and if InTEM parameters fall below cut-of values defined with ROC curves, targeted procoagulant blood components administration should be considered. MCF parameters significantly correlate to amount of CTO. Thus, ROC derived cut-off values that correspond to excessive bleeding may be used as triggers for targeted administration of procoagulant blood components.

In our study, patients in “Bleeder” category had longer CPB time (median, 116 vs. 95 min, p = 0.023) and significantly lower value of the lowest body temperature during CPB (median, 30 vs. 32 degrees of Celsius, p = 0.012). It is well known that CPB alters the hemostatic balance and predisposes cardiac surgery patients to increased risk of excessive bleeding [16]. Pathophysiology of excessive bleeding after CPB has been described by Green et al. [16]. There are several factors related to CPB that contribute to onset of hemostatic disorder such as: foreign surface contact [17], consumption of clotting factors [18], platelet activation and dysfunction [19, 20] and fibrinolysis [21]. In addition to, hemostatic impairment during CPB arises in some extent from systemic hypothermia that induces kinetic slowing of coagulation, kinin and kalikrein activation, platelet function and fibrinolysis [22]. The fact that hypothermia tends to increase bleeding in the cardiosurgical patients has been intuitively recognized by cardiac surgeons despite scarce evidence available at the beginning. Canine studies have shown that hypothermia causes thrombocytopenia [23, 24] and activates fibrinolytic system [24]. Those results were confirmed in normal volunteers both *in vitro* and *in vivo*[25] suggesting that adequate rewarming strategy may reduce the need for less safe alternative such as transfusion of procoagulant blood components.

This study has few limitations. First of all, study was prospective observational, so transfusion management was performed according to regular clinical practice. In our study the attending clinicians, both the anesthesiologists and surgeons were blinded to InTEM results, therefore administering procoagulant blood products mainly on the clinical grounds. The use of procoagulant blood products certainly affected the amount of bleeding. This decrease in amount of bleeding would reduce the degree of correlation between blood loss and both ACT and InTEM parameters by reducing the sensitivity of each parameter. Administration of procoagulant blood components certainly attenuated correlation coefficients between hemostatic test values and extent of CTO. However, it would not be ethically acceptable to evaluate the relationship between point-of-care test values and values of CTO volume amount in setting without use of procoagulant blood components administered. In our study all patients were treated in line with the best available hemostatic management. However, even if administration of procoagulant blood components distorted and attenuated correlations, the fact is that correlations were equally distorted for each test at the same patient as correlations were analyzed between CTO extent and values of respective tests. The same explanation applies for tranexaminic acid which was regularly used for all patients at two time points, (1) at the induction of anesthesia, and (2) after protamine administration. Since all patients received tranexaminic acid in the same dose and at the same time, we assume that all patients were well balanced in respect to possible effects of tranexaminic acid. In conclusion, although the transfusion of procoagulant blood components and antifibrinolytic agents certainly reduced the correlation coefficients between point-of-care test results and amount of CTO, the fact is that all patients had the same hemostatic management. Further, as previously reported within “Methods” section, patients requiring surgical reexploration for excessive bleeding were supposed to be excluded from study if bleeding vessel would be identified during reexploration. During the study period, no surgical reexploration for bleeding was performed. It is, however, obvious that CTO is consisted of both “surgical” and “hemostatic disorder” bleeding. Without surgical reexploration performed it is impossible to detect whether some proportion of patients was actually bleeding predominantly due to surgical cause. It is impossible to extract surgical bleeding from total amount of CTO. Unfortunately, it is not feasible to differentiate chest tubes bleeding volume in respect to surgical or coagulopathic origin. At our center, surgical reexploration is performed in each case with suspicion to surgical cause of bleeding based on consensus between consultant surgeon and anesthesiologist. We do not have any specific reexploration criteria based on dynamics of CTO over time as well as on amount of CTO. Without measurement of hourly CTO dynamics and clear criteria that suggest surgical bleeding it is theoretically possible that some proportion of patients had surgical bleeding even though they were not reexplorated. That may explain relatively high cut-of value for excessive bleeding in our study.

## Conclusion

In conclusion, we suggest that patients with InTEM test parameters below the cut-off values are at increased risk of excessive bleeding and use of TEM guided hemostatic management is required. Excessive bleeding with normal TEM values implies surgical bleeding and administration of procoagulant blood components such as fresh frozen plasma, fibrinogen concentrate and platelet concentrate should not simply be used empirically. Values above the cut-off values do not imply the fact that the patients will not bleed, however they may advise to pay attention to surgical cause of bleeding. Concomitant use of InTEM and HepTEM tests enables precise detection of low to moderate heparin concentration [14]. Therefore, TEM test also enables physician to detect or prevent protamine excess resulting from empirical or ACT-based additional protamine administration. Hemostatic disturbances after adequate heparin-protamine neutralization management may be easily detected with additional use of ExTEM and FibTEM assays. This may be very useful, especially in patients who developed the coagulopathy due to other CPB associated factors. In such cases, detection of coagulation factor depletion, platelets and/or fibrinogen dysfunction may lead to appropriate, “targeted” hemostatic therapy and more efficient hemostatic management. Such an approach may help to improve clinical outcome with lower incidence of excessive bleeding, lower transfusion requirements with lower incidence of transfusion related adverse outcomes.

## Authors’ original submitted files for images

Below are the links to the authors’ original submitted files for images. Authors’ original file for figure 1 Authors’ original file for figure 2 Authors’ original file for figure 3

## Abbreviations

ACT: Activating coagulation time
CPB: Cardiopulmonary bypass
CFT: Clot formation time
CT: Clotting time
CTO: Chest tube output
ECS: Elective cardiac surgery
ExTEM: Extrinsically activated thromboelastometry
FFP: Fresh frozen plasma FibTEM, extrinsically activated thromboelastometry (contains cytohalasin D)
HepTEM: Intrinsically activated thromboelastoetry (contains heparinase)
InTEM: Intrinsically activated thromboelastometry
POC: Point of care
PRBC: Packed red blood cells
ROC: Receiver operating curve
TEM: Thromboelastometry
UFH: Unfractioned heparine.
