# Supplementary figures and images for: Activated coagulation time vs. intrinsically activated modified rotational thromboelastometry in assessment of hemostatic disturbances and blood loss after protamine administration in elective cardiac surgery: analysis from the clinical trial (NCT01281397)
Source: J Cardiothorac Surg. 2014 Sep 17;9:129. doi: 10.1186/1749-8090-9-129 (PMC4283124; doi:10.1186/1749-8090-9-129)

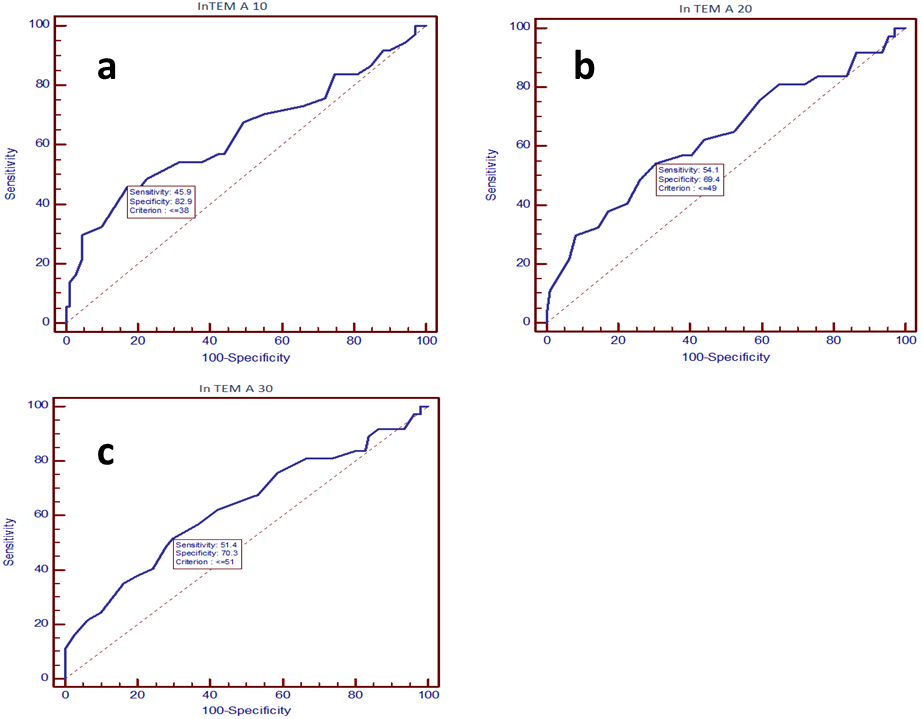

Supplement: Supplementary file 1 — Authors’ original file for figure 1 [file 13019_2013_1532_MOESM1_ESM.tiff]

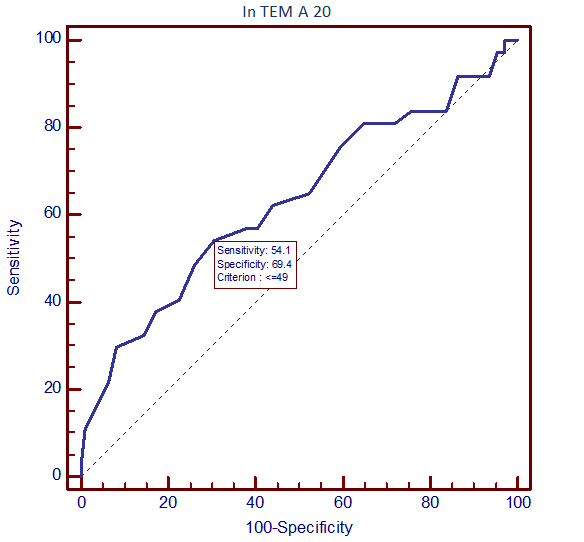

Supplement: Supplementary file 2 — Authors’ original file for figure 2 [file 13019_2013_1532_MOESM2_ESM.tiff]

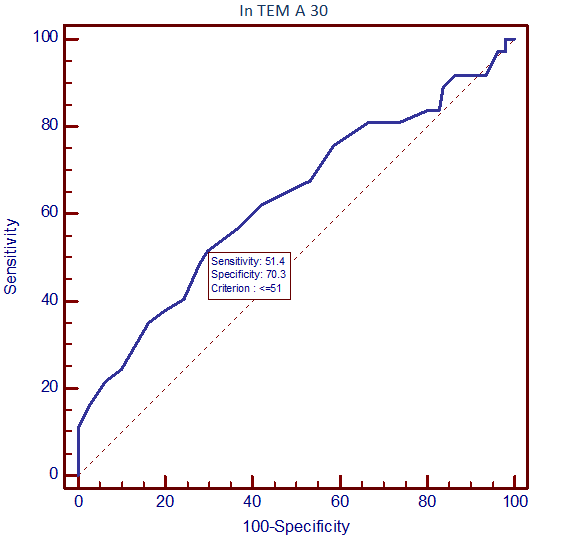

Supplement: Supplementary file 3 — Authors’ original file for figure 3 [file 13019_2013_1532_MOESM3_ESM.tiff]
